# Characteristics of tuberculosis patients at intake in Cambodia, two provinces in China, and Viet Nam

**DOI:** 10.1186/1471-2458-11-367

**Published:** 2011-05-23

**Authors:** Nguyen B Hoa, Chen Wei, Chay Sokun, Jens M Lauritsen, Hans L Rieder

**Affiliations:** 1 National Tuberculosis Programme Viet Nam, 463 Hoang Hoa Tham street, Badinh District, Hanoi, Viet Nam; 2 National Center for Tuberculosis Control and Prevention, Beijing, China; 3 National Center for Tuberculosis and Leprosy Control, Phnom Penh, Cambodia; 4 University of Southern Denmark, Institute of Public Health, Campusvej 55 DK-5230 Odense M, Denmark; 5 EpiData Association, Enghavevej 34, DK-5230 Odense, Denmark; 6 International Union Against Tuberculosis and Lung Disease, Department of Tuberculosis, 68 blvd Saint-Michel, F-75006 Paris, France; 7 University of Zurich, Institute of Social and Preventive Medicine, Hirschengraben 84 CH-8001 Zürich, Switzerland

## Abstract

**Background:**

The tuberculosis register is a critical data source for the information system of national tuberculosis control programs. From the information in the tuberculosis case register, it is possible to extend the standard analysis of age and sex characteristics among sputum smear-positive cases to all tuberculosis case categories. National tuberculosis programs might utilize such information to identify problems related to referral and access to diagnosis and treatment.

**Objectives:**

Based on the electronic database we created, our objectives were to provide a detailed description of age and sex characteristics of tuberculosis patients at registration and to provide a comparison of age-specific sex characteristics among incident and prevalent sputum smear-positive cases.

**Methods:**

A representative sample of tuberculosis case registers from 1 January 2003 to 31 December 2005 was selected in Cambodia, two provinces in China and Viet Nam. Age and sex characteristics of cases in the three separate prevalence surveys in the three jurisdictions (Cambodia: year 2002; China: year 2000; and Viet Nam: year 2006-2007) were obtained for comparison.

**Results:**

A total 37,635 patients had been registered during the period in the selected units in the three countries. Cases were more frequently male in all three countries with 53%, 71%, and 69% in Cambodia, China, and Viet Nam, respectively.

The ratios of the female-to-male odds in the notification system to that in the prevalence survey in smear-positive cases in Cambodia, China and Viet Nam were 2.1, 0.9, and 1.8, respectively. Because of the small proportion of extrapulmonary tuberculosis registered in China, we limited the analysis on age and sex distribution for extrapulmonary cases to Cambodia and Viet Nam. The proportion with extrapulmonary tuberculosis among all cases was 18.5% in Cambodia and 15.7% in Viet Nam, decreasing in frequency with increasing age.

**Conclusions:**

Characteristics of patients greatly differed between countries and between patient categories. In Cambodia and Viet Nam, efforts should be made for improved case-finding of sputum smear-positive tuberculosis among males.

## Background

The tuberculosis register is a critical data source for the information system of national tuberculosis control programs. This tool is used for routine quarterly reporting on case finding and treatment outcome.

It is of epidemiologic interest to national programs to know the characteristics of patients who are diagnosed, registered, and treated. As a routine, case finding reports in high tuberculosis burden countries include stratification by age and sex only for new (sputum) smear-positive cases as these cases have been a main program focus since the 1980s. However, from the information in the tuberculosis case register, these two major epidemiological variables may be analyzed for all tuberculosis case categories. National tuberculosis programs might use such information to identify problems related to referral and access to diagnosis and treatment.

Asia accounts for 55% of the global tuberculosis burden. The three neighboring countries, Cambodia, China, and Viet Nam that are subject of this report belong to the World Health Organization Western Pacific Region and rank respectively 21^st^, 2^nd ^and 12^th ^among the 22 high-burden countries. Our intent was to utilize a large electronic database we created from a representative sample of tuberculosis case registers from Cambodia, China, and Viet Nam to provide a more detailed description by age and sex for all major patient categories and to evaluate age- and sex-specific characteristics in the various patient categories in the three jurisdictions of our collaborative work. We further attempted to compare age-specific sex ratios among new sputum smear-positive cases in the notification system with those obtained in the prevalence surveys conducted independently from this study in the three countries. This analysis should assist clinicians and program managers to better recognize what type of patients might be expected and in which sex and age-specific groups passive case-finding as the general norm might be deficient. A comparison of notification data of incident cases with prevalent cases determined in a prevalence survey might help shedding more light on recognized sex differences in the patient population. They can provide an indication to what extent observed differences are intrinsic to the epidemiological situation and to what extent routine services might fail to find tuberculosis cases in specific age and sex groups. We focused on the two characteristics age and sex. Age is a key indicator for the epidemiologic situation as an improvement goes commonly in parallel with an increase in the age of the tuberculosis case population[1]. The distribution of cases by sex is uneven in most countries, [2] differences that have a probably complex set of explanations, including risk differences in becoming and being infected, risk differences in progression from latent infection to disease, and gender-specific differences in the probability of presenting to health facilities and being diagnosed as tuberculosis[3]. Which component is most important is likely to differ in different settings but a comparison between passively identified incident cases reflected in the notification system and actively identified prevalent cases in a survey may provide additional insight into the role of the service component as a contributor to observed sex/gender discordances.

## Methods

### Sampling

The three countries included in the study, Cambodia, China, and Viet Nam, elaborated a common research protocol to collect and analyze data from a representative sample of the routinely collected paper records of the TB case registers in the governmental sector in their respective jurisdictions[1]. Brief, in Cambodia, this roster of all management units comprised 140, in two provinces (Hubei and Jiangsu) in China 175, and in Viet Nam 668 management units. The private sector was not included in this study as appropriate sampling would have been virtually impossible to accomplish.

From each of these lists a random selection of 30 units was made and the Tuberculosis Case Register (henceforth the "case register") for two full calendar years was to be collected for computerization. The earliest permissible registration date from the selected units was 1 January 2003 and the latest 31 December 2005. Data for all tuberculosis cases registered during 2003-2004 in the selected units in Cambodia, and during the years 2004-2005 in the units selected in China and Viet Nam were extracted from the case register.

Data from the tuberculosis prevalence surveys conducted in the three jurisdictions [4-6] on sputum smear-positive cases by age and sex were obtained though the three respective national tuberculosis programs.

### Study approval

In Cambodia, study approval was obtained from the Director of the National Center for Tuberculosis and Leprosy Control, in China from the Director of the National Center for Tuberculosis Control and Prevention, and in Viet Nam from the Director of the Viet Nam National Tuberculosis Control Program. Ethical approval for the study was obtained from The Union Ethics Advisory Committee.

### Data entry and validation

The electronic data collection instrument was prepared with EpiData Entry (Version 3.1, freely available at http://www.epidata.dk). All information from the case register, except for the patient name and address was captured by double-entry, entering all data twice and independently into two datasets. The two putatively identical datasets were compared electronically and any undiscovered discordance between the two datasets resulting from double-entry was resolved by rechecking against the original case register and making the necessary corrections in a final dataset that was subsequently used for analysis.

### Data analysis

All analyses were done using EpiData Analysis (Version 2.2, freely available at http://www.epidata.dk). The odds ratio of comparison to prevalence surveys was calculated by dividing the odds of female to male cases in the tuberculosis register by the odds of female to male cases in the respective three prevalence surveys, (Cambodia year 2002, China year 2000, and Viet Nam year 2006-2007).

The definition of tuberculosis patient categories followed those described in the "Revised international definitions in tuberculosis control"[7].

## Results

### Characteristics of patients at intake of the tuberculosis case register in Cambodia, China and Viet Nam

In each country, all 30 pre-determined, randomly selected case registers were successfully obtained and captured electronically. The final total dataset comprised 37,635 patients for analysis, i.e. 4,215 patients (11.2%) from Cambodia, 26,257 patients (69.8%) from China, and 7,163 patients (19.0%) from Viet Nam.

Table 1 shows the characteristics of the 37,635 patients at registration in the case register in the three study countries. Of these cases, 20.3% were among patients aged 65 years or older. The proportion among all patients who were 65 years old and older was higher in China and Viet Nam compared with Cambodia (21.5% and 20.6% vs. 12.6%, respectively). The proportion of males was higher in China and Viet Nam compared to Cambodia (71% and 69% vs. 53%, respectively).

**Table 1:** Characteristics of patients at intake in the Tuberculosis case register, Cambodia, China (Hubei and Jiangsu provinces), and Viet Nam

Characteristic	Cambodia	China	Viet Nam	Total
	n; (%)*	n; (%)*	n; (%)*	n; (%)*
**Total**	**4215 (100)**	**26257 (100)**	**7163 (100)**	**37635 (100)**
**Age group**				
0- to 14-yr-old	103(2.4)	173 (0.7)	84 (1.2)	360 (1.0)
15- to 24-yr-old	407 (9.7)	3601 (13.7)	737 (10.3)	4745 (12.6)
25- to 34-yr-old	632 (15.0)	3906 (14.9)	1219 (17.0)	5757 (15.3)
35- to 44-yr-old	974 (23.1)	4280 (16.3)	1330 (18.6)	6584 (17.5)
45- to 54-yr-old	856 (20.3)	4351 (16.6)	1446 (20.2)	6653 (17.7)
55- to 64-yr-old	709 (16.8)	4296 (16.4)	873 (12.2)	5878 (15.6)
65 years and older	533 (12.6)	5650 (21.5)	1472 (20.6)	7655 (20.3)
Age not recorded	1	0	2	3
**Sex**				
Female	1981 (47.0)	7565 (28.8)	2200 (30.7)	11746 (31.2)
Male	2233 (53.0)	18686 (71.2)	4962 (69.3)	25881 (68.8)
Sex not recorded	1	6	1	8
**Site**				
Pulmonary	3434 (81.5)	25849 (98.8)	6035 (84.3)	35318 (94.1)
Extrapulmonary	781 (18.5)	302 (1.2)	1122 (15.7)	2205 (5.9)
No site recorded	0	106	6	112
**Category**				
New	3901 (92.8)	21913 (84.0)	6012 (87.9)	31826 (85.7)
Relapse	192 (4.6)	1845 (7.1)	523 (7.6)	2560 (6.9)
Treatment after failure	11 (0.3)	35 (0.1)	64 (0.9)	110 (0.3)
Treatment after default	5 (0.1)	454 (1.7)	26 (0.4)	485 (1.3)
Transfer in	79 (1.9)	2 (0.0)	155 (2.3)	236 (0.6)
Other	16 (0.4)	1832 (7.0)	58 (0.8)	1906 (5.1)
Category not recorded	11	176	325	512

* Column percent

The proportion of cases with extrapulmonary tuberculosis was very low in China (1.2%) compared with Viet Nam (15.7%) and Cambodia (18.5%).

### Age and sex stratification by patient category

The highest proportion of female patients was found among extrapulmonary cases (45.4%), while the proportion of female cases among all cases was substantially lower and similar among both new smear-positive and new smear-negative pulmonary cases (30.9% and 30.6% respectively). The proportion was lowest among smear-positive re-treatment cases (relapse, treatment after failure and treatment after default) and the categories "other" and transfer in (the latter two combined) with 27.3% and 27.8% respectively (table 2).

**Table 2:** Five categories by sex and age group in the Tuberculosis case register, Cambodia, China (Hubei and Jiangsu provinces), and Viet Nam

Characteristic	New pulm sm-pos	Re-tx sm-pos	Extrapulmonary	New pulm sm-neg	All other/transfer in	Total
	n; (%)*	n; (%)*	n; (%)*	n; (%)*	n; (%)*	n; (%)*
**Total**	22335 (100)	2631 (100)	2205 (100)	7786 (100)	2678 (100)	37635 (100)
**Sex**						
Female	6905 (30.9)	719 (27.3)	1000 (45.4)	2379 (30.6)	743 (27.8)	11746 (31.2)
Male	15425 (69.1)	1911 (72.7)	1205 (54.6)	5406 (69.4)	1934 (72.2)	25881 (68.8)
No sex recorded	5	1	0	1	1	8
**Age group**						
0- to 14-yr-old	109 (0.5)	2 (0.1)	141 (6.4)	93 (1.2)	15 (0.6)	360 (1.0)
15- to 24-yr-old	3095 (13.9)	117 (4.4)	370 (16.8)	893 (11.5)	270 (10.1)	4745 (12.6)
25- to 34-yr-old	3568 (16.0)	270 (10.3)	498 (22.6)	1100 (14.1)	321 (12.0)	5757 (15.3)
35- to 44-yr-old	4022 (18.0)	457 (17.4)	439 (19.9)	1180 (15.2)	486 (18.1)	6584 (17.5)
45- to 54-yr-old	3942 (17.7)	567 (21.6)	326 (14.8)	1257 (16.1)	561 (20.9)	6653 (17.7)
55- to 64-yr-old	3366 (15.1)	537 (20.4)	207 (9.4)	1240 (15.9)	528 (19.7)	5878 (15.6)
65 years and older	4232 (18.9)	681 (25.9)	223 (10.1)	2022 (26.0)	497 (18.6)	7655 (20.3)
No age recorded	1	0	1	1	0	3

* Column percent

Pulm sm-pos: Pulmonary smear-positive; Re-tx sm-pos: Retreatment smear-positive; New pulm sm-neg: new pulmonary smear-positive.

### Age and sex distribution in new smear-positive tuberculosis cases

**Figure 1:**
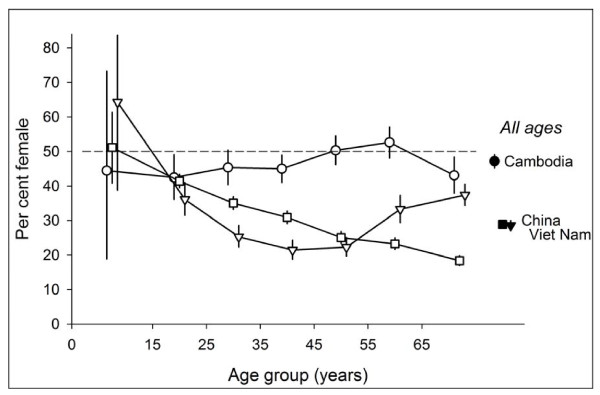
**New smear-positive in the tuberculosis case register, Cambodia, China (Hubei and Jiangsu provinces), and Viet Nam, by sex and country**.

Figure 1 shows the proportion of female patients among new sputum smear-positive tuberculosis cases, by age and country. The proportion of females among new sputum smear-positive tuberculosis cases in China (28.9%) decreased with increasing age. While this proportion was fairly stable in Cambodia across age groups, it was lowest in the age group of the 15- to 24-year-old (42.5%) and highest in the 55- to 64-year-old age group (52.6%). The proportion of females among new smear-positive cases in Viet Nam (28.6%) decreased from the 0- to 14-year-old age group (64.3%) to the 35- to 44-year-old age group (21.5%) and increased above that age slightly in the 45- to 54-year-old age group (22.2%) to the age group 65 years and older (37.4%).

### Age-specific sex ratio of new smear-positive tuberculosis cases in the tuberculosis register compared with prevalence surveys

**Figure 2:**
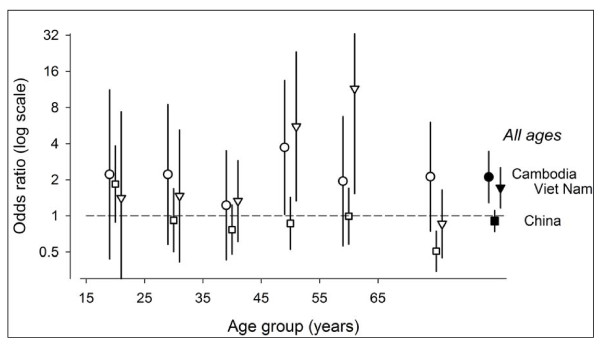
**Odds ratio of being notified as new female smear-positive case compared to being a female smear-positive case in the prevalence survey, in Cambodia, China (Hubei and Jiangsu provinces), and Viet Nam**.

Figure 2 shows the odds ratio of being notified as a new female smear-positive case compared to being a female smear-positive case in the prevalence survey in Cambodia, China, and Viet Nam were 2.1 (95% CI: 1.3-3.5), 0.9 (95% CI: 0.8-1.1), and 1.8 (95% CI: 1.2-2.6), respectively. Among the three countries, this ratio was largest in Cambodia and ranged from 1.9 to 2.2 between age groups (except for the age group 35- to 44-year-old (1.2) and 45- to 54-year-old (3.7)). The odds ratio was close to unity in China and ranged from 0.8 to 1 in different age groups, except for the age group of 65 years old and older (0.5) and the 15- to 24-year-old (1.8). In Viet Nam, this ratio ranged from 0.9 to 1.5 in different age groups, except in the age group of the 45- to 54-year-old (5.6) and the 55- to 64-year-old (11.5).

### Age and sex distribution among extrapulmonary cases

**Figure 3:**
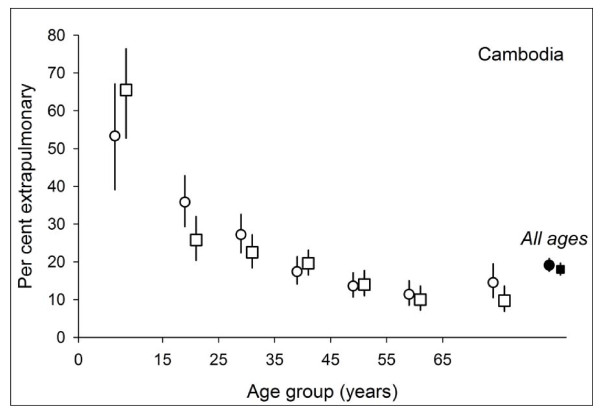
**Proportion of extrapulmonary tuberculosis among all cases, by age and sex in Cambodia.** Circles indicate female, squares male patients.

**Figure 4:**
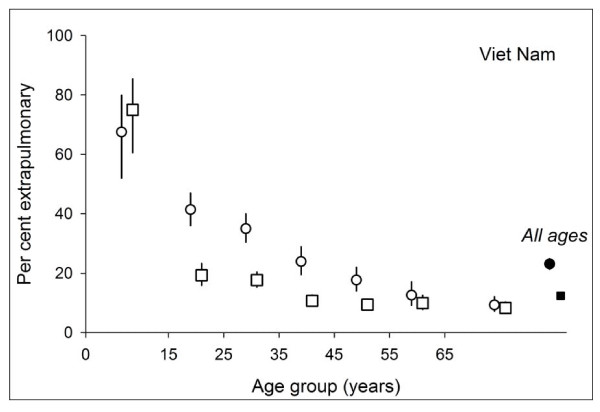
**Proportion of extrapulmonary tuberculosis among all cases, by age and sex in Viet Nam.** Circles indicate female, squares male patients.

Because of the small proportion of extrapulmonary cases (1.2%) registered in China, we limited this part of the analysis to Cambodia and Viet Nam. Figure 3 shows the proportion of extrapulmonary cases among all cases by sex and age in Cambodia (Figure 3) and Viet Nam (Figure 4).

In Cambodia, the proportion of extrapulmonary cases among all cases decreased by age group in both males and females. The proportion of extrapulmonary was not different between males (18.0%, 95% CI: 16.5-19.7) and females (19.1%, 95% CI: 17.5-20.9).

Similarly, in Viet Nam, the proportion of extrapulmonary among all cases who were female also decreased with increasing age. In contrast, among males, this proportion was quite stable in the four examined age groups of those 35 years and older (10.7%, 9.4%, 9.9%, 8.3%, respectively). The proportion of extrapulmonary was higher among female (23.1%) than among male cases (12.4%).

**Figure 5:**
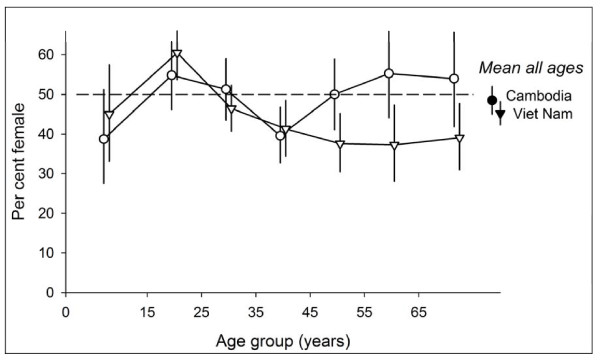
**Proportion of female among extrapulmonary tuberculosis, by age, Cambodia and Viet Nam**.

Figure 5 shows the proportion of females among extrapulmonary tuberculosis cases by age group in Cambodia and Viet Nam. The proportion of females among extrapulmonary cases was 48.5% in Cambodia and 45.2% in Viet Nam.

## Discussion

We found significant differences in the distribution of tuberculosis cases by sex and age groups in all three countries. Cases were more frequently male in all tuberculosis categories, and in all three countries. A previous study shows that the female-to-male prevalence ratios were less than 0.5 in surveys in the South-East Asia and Western Pacific region, and approximately 1 in the African Region[2]. The information on sex and age groups in all tuberculosis categories is not available in standard WHO routine reports. It is however known to vary importantly by age, sex, and country of origin from several analyses that had examined it more closely[2,8-13].

We also found that the proportion with extrapulmonary tuberculosis among all cases was decreasing in frequency with increasing age.

In all three countries, three quarters of patients occurred among adolescents and adults aged less than 65 years, and one fifth among patients aged 65 years or more. The distribution by age in tuberculosis patients seems to be similar with this figure in 32 European countries reported in the year 1995[14]. A rising age in tuberculosis is regarded as an epidemiologically encouraging sign as it reflects a decrease in infection risk over calendar time with a resulting shift of the bulk of the prevalence of prior acquired infection to older age groups from which cases continue to emerge[3]. That the proportion of cases in the age group 65 years and above was lowest in Cambodia compared to China and Viet Nam thus might indicate that the tuberculosis epidemiology in Cambodia is still larger than in the two other countries. This information is also consistent with the results of the three prevalence surveys which showed that the prevalence of smear-positive tuberculosis was higher in Cambodia (269 per 100,000 population) than in Viet Nam (145 per 100,000) and China (122 per 100,000)[4-6]. The incidence estimates for Cambodia are also higher than those for Viet Nam and China[15]. The relationship between the proportion of tuberculosis cases in the oldest age group of the 65-year-old and older population and the findings from prevalence surveys would deserve a more thorough investigation to ascertain to what extent such an indicator might be used to assess the epidemiology of tuberculosis.

The male-to-female ratio in China and Viet Nam was more than 2, a common observation in many countries[4,11]. In contrast, in Cambodia there was roughly a balance of cases among the two sexes. Differences in the sex ratio can be attributed to the risk of becoming and being infected, risk differences in progression from latent infection to overt clinical tuberculosis, and differences in accessibility to health services[3]. Male-female differences in the prevalence of infection with *Mycobacterium tuberculosis *are frequently small up to adolescence while subsequently the risk of becoming infected with *M. tuberculosis *and hence the resulting prevalence of infection is frequently higher among males than among females[3]. However, the risk of progression from latent infection to tuberculosis varies greatly by age and has commonly been found to be larger among females than males in young adults while this changes to the opposite with increasing age[16-19]. As these two findings run in the opposite direction, one thus might expect that the contribution of females to the total number of cases to be larger where tuberculosis occurs among the young. That this is indeed the case has been shown in Denmark for example where the male-to-female ratio increased steadily over time in parallel with an improvement of the epidemiologic situation and a shift of the age structure of tuberculosis patients to older age groups[16].

The low proportion of extrapulmonary tuberculosis reported in China finds perhaps largely an explanation in the fact that the treatment for extrapulmonary tuberculosis, in contrast to sputum smear-positive tuberculosis, is not offered free of charge and patients thus may seek care outside the governmental clinics captured in this sample.

Extrapulmonary tuberculosis is virtually always and only based on a clinical decision and rarely supported by bacteriological findings. In all our settings the diagnosis of extrapulmonary tuberculosis must be made by a qualified clinician.

In this study, we observed a substantial decrease in the proportion of extrapulmonary among all cases with age, clearly among both male and female patients in Cambodia and clearly among female patients in Viet Nam. That the proportion of extrapulmonary tuberculosis among all patients decreased with increasing age had also been observed in other studies[8,20].

We observed the proportion of extrapulmonary among all cases to be higher among females than among males, a finding similar to that reported in other studies[21-23]. That extrapulmonary tuberculosis disproportionately affects females is a well-known observation [8,22,23,23]. Extrapulmonary tuberculosis is also associated with young age[8,22]. This can explain, at least partially, the larger female proportion in Cambodia compared to Viet Nam. Nevertheless, failure of diagnosing extrapulmonary tuberculosis, particularly unusual manifestations, might be of concern in many national programs and needs to be better investigated. That the proportion of extrapulmonary tuberculosis was higher in Cambodia than in Viet Nam might also find a contributory explanation in the higher prevalence of HIV infection in Cambodia than Viet Nam[15].

A comparative analysis of ratios between notification data and the prevalence survey data has the strength that it can potentially unravel relative differences resulting from other factors as the comparative measure does not depend on population figures.

In comparison with the prevalence surveys we found that the ratios of being notified as a new female smear-positive case compared to being a female smear-positive case in the prevalence survey were 2.1, 0.9 and 1.8 in Cambodia, China and Viet Nam. This suggests that males, compared to females, were under-diagnosed in Cambodia and Viet Nam. If this hypothesis is correct, then the national tuberculosis programs in Cambodia and Viet Nam need to pay more attention to case finding among males. This observation is also consistent with the conclusion from the prevalence survey in Cambodia in the year 2000 which noted that case detection among males must be much lower than that among females, [4] an observation also reported from the prevalence survey conducted in Viet Nam in the years 2006-2007. The patient diagnostic rate, a program performance measure proposed by Borgdorff, [24] is the rate at which prevalent cases are detected by a control program, and it was higher in women compared to men in the survey in Viet Nam[6].

An important limitation of our study is that it did not cover patients treated in the private sector and those parts of the public sector failing to report to the NTP. It is conceivable that there may be differences in sex and age among such patients compared to the public sector reporting to NTPs. Another limitation lies with the impossibility to synchronize the study period with the time period covered by the prevalence survey. To the extent the tuberculosis prevalence had changed unequally for age and sex over time our analysis would have been biased. However, the time difference between the prevalence surveys and the notification data captured in our analysis is rather small and such a bias might thus be of minor importance.

## Conclusions

Notable differences in the characteristics of the sex/age composition of all tuberculosis patients in general and among extrapulmonary cases specifically have been observed between the countries. In Cambodia and Viet Nam this may point to possible gains associated with efforts for diagnosis of smear-positive tuberculosis in males. With the increased availability of computers at all levels of the health system, an increasing number of countries has been initiating or is planning to use electronic recording and reporting systems. Where such computerization has been implemented, a systematic and routinely performed analysis by age and sex for all patient categories must be given strong consideration. It should potentially permit providing more information about and for the tuberculosis programs.

## Competing interests

The authors declare that they have no competing interests.

## Authors' contributions

All the authors contributed to the design of the study. NBH, CS, CW were responsible for the implementing the study in the countries, data collection and data entry. HLR was responsible for the conception and overall supervision of the quality data collection, data entry and analysis. NBH, HLR, JML were responsible for data analysis. NBH and HLR wrote the first draft of the paper and all co-authors contributed to the writing of the final paper. All authors read and approved the final manuscript. HLR is guarantor of the paper.

## Pre-publication history

The pre-publication history for this paper can be accessed here:

http://www.biomedcentral.com/1471-2458/11/367/prepub
